# Impaired cerebro-cerebellar white matter connectivity and its associations with cognitive function in patients with schizophrenia

**DOI:** 10.1038/s41537-021-00169-w

**Published:** 2021-08-12

**Authors:** Sung Eun Kim, Sungcheol Jung, Gyhye Sung, Minji Bang, Sang-Hyuk Lee

**Affiliations:** 1 Department of Psychiatry, CHA Bundang Medical Center, CHA University, Seongnam, Republic of Korea; 2 CHA University School of Medicine, Seongnam, Republic of Korea; 3 Department of Psychology, Korea University, Seoul, Republic of Korea

**Keywords:** Schizophrenia, Biomarkers, Neural circuits

## Abstract

Schizophrenia is a complex brain disorder of unknown etiology. Based on the notion of “cognitive dysmetria,” we aimed to investigate aberrations in structural white matter (WM) connectivity that links the cerebellum to cognitive dysfunction in patients with schizophrenia. A total of 112 participants (65 patients with schizophrenia and 47 healthy controls [HCs]) were enrolled and underwent diffusion tensor imaging. Between-group voxel-wise comparisons of cerebellar WM regions (superior/middle [MCP]/inferior cerebellar peduncle and pontine crossing fibers) were performed using Tract-Based Spatial Statistics. Cognitive function was assessed using the Trail Making Test Part A/B (TMT-A/B), Wisconsin Card Sorting Test (WCST), and Rey-Kim Memory Test in 46 participants with schizophrenia. WM connectivity, measured as fractional anisotropy (FA), was significantly lower in the MCP in participants with schizophrenia than in HCs. The mean FAs extracted from the significant MCP cluster were inversely correlated with poorer cognitive performance, particularly longer time to complete the TMB-B (*r* = 0.559, *p* < 0.001) and more total errors in the WCST (*r* = 0.442, *p* = 0.003). Our findings suggest that aberrant cerebro-cerebellar communication due to disrupted WM connectivity may contribute to cognitive impairments, a core characteristic of schizophrenia. Our results may expand our understanding of the neurobiology of schizophrenia based on the cerebro-cerebellar interconnectivity of the brain.

## Introduction

Schizophrenia is a complex brain disorder of unknown etiology. Neurobiological models of schizophrenia have focused on structural and functional abnormalities in the cerebrum, particularly the fronto-temporo-limbic areas^1,2^; however, the understanding of schizophrenia remains unsatisfactory because none of the proposed models fully explain its pathogenetic mechanisms. Therefore, demand for the development of a new, integrated neurobiological model for schizophrenia is increasing.

Cognitive dysfunction is a key feature of schizophrenia; it emerges prior to the onset of full-blown psychosis and persists after the remission of psychotic symptoms^3,4^. Since the “cognitive dysmetria” model of schizophrenia was first introduced^5^, the cerebellum has been implicated in the cognitive dysfunction of patients with schizophrenia. Cognitive dysmetria refers to the slippage of the cerebellum in modulating and coordinating multimodal information conveyed from the cerebral cortex, resulting in erroneous cognition and behaviors^6^. Anatomical evidence shows that the cerebellum is connected to the prefrontal cortex through polysynaptic closed-loop circuits; from the prefrontal cortex, neuronal fibers are projected to the cerebellum via the pons, while cerebellar projections return to the cortex via the thalamus^7^. In higher-order cognitive function, the cerebellum has been suggested to parallel its role in the fine control of motor movements to regulate the speed, balance, coordination, and accuracy^8^. This is supported by early reports describing impairments to executive function, visuospatial organization, and language processing, as well as inappropriate effect and behavior in patients with acquired lesions in the cerebellum^9^. These cognitive-affective symptoms overlap with those observed in patients with schizophrenia, implying the possible involvement of the cerebellum in the development of schizophrenia.

The cerebellum communicates with the other central nervous system through the three major white matter (WM) bundles: superior (SCP), middle (MCP), and inferior (ICP) cerebellar peduncles^10^. The SCP and MCP form the cortico-ponto-cerebello-thalamo-cortical tract to interconnect the cerebrum and cerebellum. The SCP contains efferent fibers that emerge from the deep cerebellar nuclei, pass through the ipsilateral SCP into the dorsal pons, and travel to the thalamus, followed by the contralateral cerebral cortex. The MCP, the largest structure among the cerebellar WM tracts, is a major afferent pathway that receives input signals from the contralateral cerebral cortex via pontine nuclei. Conversely, the ICP transfers input signals from the olivary nucleus and spinal cord into the cerebellum and sends cerebellar output signals to the vestibular nucleus. These cerebellar peduncles have an essential role in integrating the cerebellum into distributed neural systems to modulate and optimize cognitive, affective, and sensorimotor activities through trial-and-error learning^10,11^.

Although the majority of studies have focused primarily on supratentorial structures, emerging evidence has shed light on structural and functional disruptions in cerebro-cerebellar connectivity in patients with schizophrenia^12–16^. A functional magnetic resonance imaging (MRI) study showed that cerebro-cerebellar functional connectivity within the default-mode network was decreased in individuals at ultra-high risk for psychosis and with first-episode schizophrenia compared to that in healthy controls (HCs)^12^. Structurally, diffusion tensor imaging (DTI) studies demonstrated that patients with schizophrenia had smaller fractional anisotropy (FA) in the SCP^15^ and MCP^14,16^ compared to HCs and higher FAs of the left SCP were correlated with poorer cognitive functioning measures using the cognitive cluster scores from the Positive and Negative Syndrome Scale (PANSS)^15^. In a tractography study, FA deficits with normal mean diffusivity (MD) were found across all cerebro-cerebellar and intra-cerebellar WM tracts, suggesting their architectural disorganization in patients with schizophrenia^13^. However, those previous studies compared averaged DTI measures within each region of interest (ROI), which limited the power to detect small, focal deficits, and the sample sizes were relatively small. Recently, a multisite mega-analysis of 983 patients and 1349 HCs revealed that cerebellar volumes are robustly reduced in patients with schizophrenia compared to HCs and positively correlated with cerebral cortical thickness in frontotemporal regions^17^. Although this study did not assess the direct connectivity between the cerebrum and cerebellum, its results with a large dataset imply that the cerebellum and cerebrum would be affected by common pathogenetic processes of schizophrenia in an interrelated manner. Hence, taken together, it is necessary to investigate cerebro-cerebellar WM connectivity as one of the potential markers of abnormality associated with cognitive dysfunction in patients with schizophrenia.

This study sought to investigate the structural connectivity of the three major cerebellar WM bundles in relation to cognitive dysfunction in patients with schizophrenia. We hypothesized that WM connectivity would be altered in patients with schizophrenia in comparison with HCs. We assumed that the SCP and MCP were more likely to be damaged in patients with schizophrenia as these two regions contain polysynaptic fibers interconnecting the cerebrum and cerebellum. Second, we hypothesized that the cognitive performance of patients with schizophrenia would be associated with WM connectivity in the WM regions showing aberrant cerebro-cerebellar connectivity in comparison with HCs. Given the fine-tuning role of the cerebellum in mental processing and motor behaviors, we suggest that the performance of executive function would demonstrate the strongest association.

## Results

### Demographics and cognitive function of the study participants

Table 1 summarizes the demographic and clinical characteristics of participants with schizophrenia and HCs. There were no significant differences between participants with schizophrenia and HCs in demographic variables except for years of education. Fifty-six (86.2%) of the 65 participants with schizophrenia were recent-onset patients who had developed psychotic symptoms within the last 5 years. Neuroimaging data were acquired within 4 weeks of the initiation of antipsychotic treatment if participants were uncooperative because of exacerbated psychotic symptoms. The descriptive data for the measures of cognitive function in participants with schizophrenia are presented in Table 2 (*n* = 46). The characteristics of participants with schizophrenia who completed the cognitive assessment are presented in Supplementary Table 1. The cognitive performance was not correlated with either the duration of illness (∣*r*∣ < 0.093, *p* > 0.568) or chlorpromazine equivalent dose (∣*r*∣ < 0.234, *p* > 0.117). Age, sex, and years of education were controlled for as potential confounders for WM connectivity and cognitive function in the subsequent analysis.

**Table 1:** Demographic and clinical characteristics of study participants.

	Schizophrenia (*n* = 65)	HCs (*n* = 47)	Statistics	*p* value
Sex				
Male, *n* (%)	26 (40.0)	24 (51.1)	*χ*^*2*^ = 1.35	0.245
Female, *n* (%)	39 (60.0)	23 (48.9)		
Age (years, mean ± SD)	34.4 ± 8.6	36.7 ± 8.6	*t* = −1.40	0.164
Education (years, mean ± SD)	12.9 ± 2.9	17.2 ± 2.0	*t* = −9.25	<0.001
Duration of illness (months, mean ± SD)	17.7 ± 33.4			
Smoking, *n* (%)	23 (35.4)	10 (21.3)	*χ*^*2*^ = 2.62	0.106
Alcohol, *n* (%)	17 (26.2)	15 (31.9)	*χ*^*2*^ = 0.44	0.505
Antipsychotic exposure				
Naïve, *n* (%)	60 (92.3)			
> 6 months free, *n* (%)	5 (7.7)			
Other psychotropic medication				
Mood stabilizer, *n* (%)^a^	2 (3.1)			
Antidepressant, *n* (%)^b^	4 (6.2)			
Duration of antipsychotics before MRI scan (days, mean ± SD)	6.5 ± 7.1			
Chlorpromazine equivalent dose of antipsychotics at MRI scan (mg/day, mean ± SD)	455.0 ± 260.5			
CGI-S (mean ± SD)	5.0 ± 1.0			
PANSS (mean ± SD)				
Positive symptom	31.1 ± 6.6			
Negative symptom	25.1 ± 8.7			
General psychopathology	62.5 ± 12.4			

*HCs* healthy controls, *SD* standard deviation, *MRI* magnetic resonance imaging, *CGI-S* Clinical Global Impression—Severity, *PANSS* Positive and Negative Syndrome Scale.

^a^All participants receiving mood stabilizer were taking divalproex sodium.

^b^Antidepressants administered by participants were sertraline (*n* = 2) and escitalopram (*n* = 2).

**Table 2:** Cognitive performance in participants with schizophrenia (*n* = 46).

	Mean ± SD
Trail-making test (seconds)	
Part A	52.1 ± 48.8
Part B	108.4 ± 62.6
Wisconsin card sorting test (raw score)	
Total errors	49.7 ± 26.3
Perseverative responses	25.7 ± 17.1
Perseverative errors	22.5 ± 13.9
Non-perseverative errors	27.2 ± 18.0
Conceptual level responses	47.7 ± 23.4
Rey–Kim memory test (raw score)	
Verbal memory: free recall 1–5	38.7 ± 12.2
Verbal memory: delayed recall	6.6 ± 3.5
Verbal memory: delayed recognition	11.8 ± 3.2
Visual memory: copy	30.9 ± 7.5
Visual memory: immediate recall	15.2 ± 7.7
Visual memory: delayed recall	14.0 ± 7.5

### Voxel-wise comparison of FAs in cerebellar peduncles between participants with schizophrenia and HCs

**Fig. 1:**
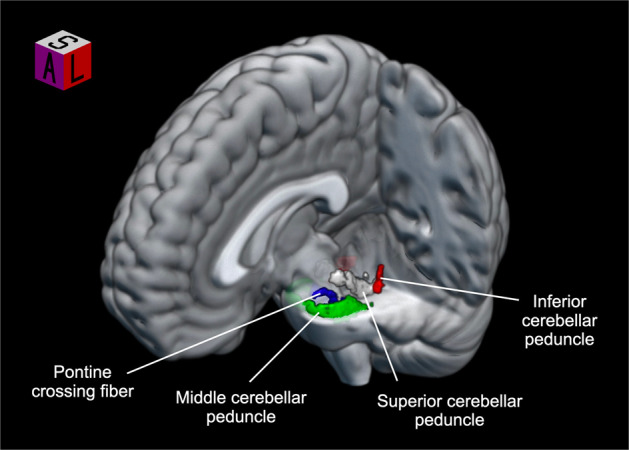
White matter regions of interest. Major white matter bundles connecting the cerebellum to other central nervous system were chosen based on the JHU DTI-based white matter atlas. The region-of-interest mask was created by multiplying the mean FA skeleton mask with the regional mask of the cerebellar peduncle.

**Fig. 2:**
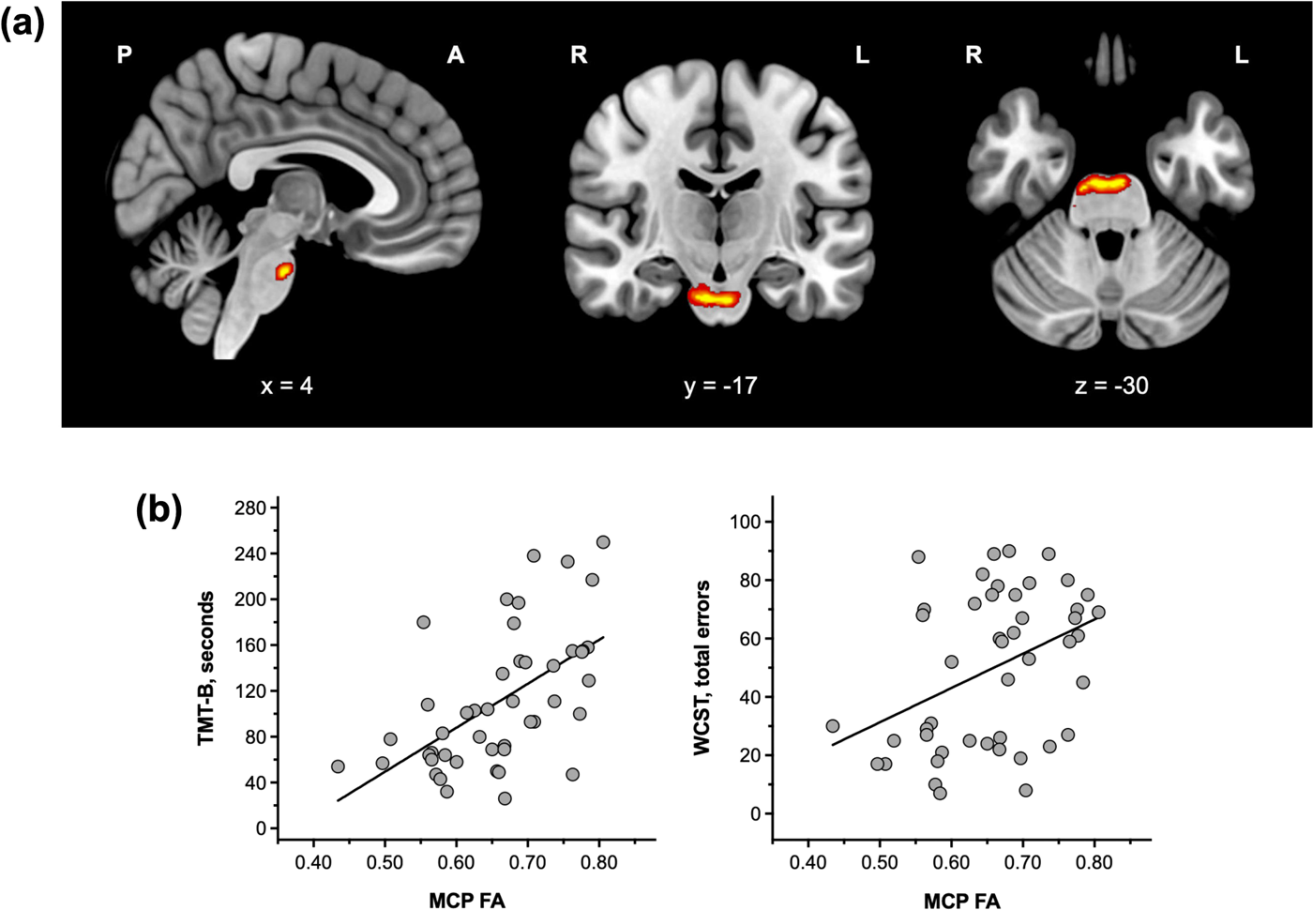
Between-group comparison of cerebro-cerebellar white matter connectivity and relationship between MCP FAs and cognitive performance in participants with schizophrenia. **a** Participants with schizophrenia showed significantly lower FAs in a cluster located in the middle cerebellar peduncle compared to healthy controls (TFCE-corrected *p* < 0.05). **b** Higher MCP FAs were associated with a longer time taken to complete the TMT-B and more total errors in the WCST in participants with schizophrenia (*n* = 46).

The ROIs were defined based on the Johns Hopkins University (JHU) DTI-based WM atlas (Fig. 1)^18^. Voxel-wise comparison, controlled for age, sex, and years of education, revealed that FAs were significantly lower in the MCP in participants with schizophrenia compared to HCs (*p* < 0.05, threshold-free cluster enhancement [TFCE] corrected; Fig. 2a). No significant differences were found between the two groups for MD, axial diffusivity (AD), and radial diffusivity (RD) maps. The mean FAs were extracted from the significant cluster to conduct a correlation analysis (mean ± standard deviation [SD]; schizophrenia: 0.66 ± 0.09; HCs: 0.72 ± 0.09).

### Relationship between FA in the MCP and cognitive performance in participants with schizophrenia

The correlation analysis demonstrated that higher mean FAs in the MCP cluster were significantly associated with poorer performance in the Trail Making Test, Part B (TMT-B), Wisconsin Card Sorting Test (WCST), Auditory Verbal Learning Test (AVLT), and Complex Figure Test (CFT) after controlling for age, sex, and years of education (Table 3). Participants with schizophrenia who had higher mean FAs in the MCP took longer to complete the TMT-B and made more errors on the WCST. For memory function, FAs in the MCP were inversely correlated with the performance in the free and delayed recall trials on the AVLT and the copy trial on the CFT. The correlations with MCP FAs were statistically significant after Bonferroni correction for the time spent on the TMT-B and number of total errors on the WCST (*p* < 0.05/13 = 0.004; Fig. 2b). The statistical significance of the results remained grossly unchanged after controlling further for the duration of illness and chlorpromazine equivalent dose.

**Table 3:** Correlations between FAs in the MCP cluster and cognitive performance in participants with schizophrenia (*n* = 46).

	MCP FAs^a^
Trail making test	
Part A	*r* = 0.083, *p* = 0.615
Part B	*r* = 0.559, *p <* 0.001^b^
Wisconsin card sorting test	
Total errors	*r* = 0.442, *p* = 0.003^b^
Perseverative responses	*r* = 0.312, *p* = 0.041
Perseverative errors	*r* = 0.336, *p* = 0.027
Non-perseverative errors	*r* = 0.370, *p* = 0.014
Conceptual level responses	*r* = 0.395, *p* = 0.009
Rey–Kim memory test	
Verbal memory: free recall 1-5	*r* = −0.365, *p* = 0.016
Verbal memory: delayed recall	*r* = −0.387, *p* = 0.010
Verbal memory: delayed recognition	*r* = −0.299, *p* = 0.052
Visual memory: copy	*r* = −0.317, *p* = 0.036
Visual memory: immediate recall	*r* = −0.225, *p* = 0.142
Visual memory: delayed recall	*r* = −0.281, *p* = 0.064

*FA* fractional anisotropy, *MCP* middle cerebellar peduncle.

^a^Controlled for age, sex, and years of education as covariates.

^b^Remained statistically significant after Bonferroni correction.

## Discussion

The present study investigated structural abnormalities in WM tracts interconnecting the cerebrum and cerebellum and their association with cognitive function in participants with schizophrenia. We demonstrated that participants with schizophrenia had significantly lower FAs in the MCP than did HCs and showed an inverse correlation between FAs in the MCP and performance in executive function measured by the TMT-B and WCST. Our findings suggest that WM dysconnectivity in afferent fibers from the cerebrum to the cerebellum may be associated with impaired cognitive function in patients with schizophrenia, which is consistent with the notion of cognitive dysmetria.

The finding of lower FAs in the MCP of patients is grossly consistent with previous studies showing the aberrant microstructural organization of cerebellar WM tracts in schizophrenia^13–16,19^. The MCP is the major afferent fiber bundle that connects the cerebellum and contralateral cerebral cortex via pontine nuclei^10^ and conveys vast amounts of sensory, motor, and associative information from the cerebrum to cerebellum^20^. DTI-assessed FA is a sensitive measure of the degree of water diffusion along the fiber tracts, determined by structural features of the myelin sheath, axonal membrane, and neurofibrils^21^. Since FA is affected by water diffusivity parallel (AD) and/or perpendicular (RD) to WM tracts, FA changes are generally accompanied by alterations in MD, AD, and RD^22,23^. However, as demonstrated by our results, lower FAs have been observed with no differences in diffusivity measures compared to HCs in patients with psychiatric disorders, including schizophrenia^13,24–26^. It is well-known that AD is associated with axonal density or caliber, whereas RD is related to myelin compactness. Although none of the DTI measures are specific to certain WM pathology^27^, our current findings may suggest that the breakdown of complex neuronal architecture in the MCP, rather than simple demyelination or axonal damage, is associated with the pathogenesis of schizophrenia.

The involvement of the MCP is common in various neurodegenerative and demyelinating diseases^28^. WM hyperintensity of the MCP is associated with more severe cognitive deficits and a longer history of symptoms in patients with fragile X-associated tremor/ataxia syndrome^29^. Although WM signal intensity of the MCP is within the normal range for patients with schizophrenia, subtle and microscopic changes in this region may cause impaired signal transmission via the cortico-ponto-cerebellar tract. Since this tract contains fibers forming a closed loop between the cerebellum and precentral/prefrontal cortex^30^, WM aberrations in the MCP may hinder the cerebellum from providing adaptive feedback in response to cerebral inputs for various cognitive processes. Although we cannot determine whether WM abnormalities in the MCP is a primary cause of schizophrenia in this cross-sectional study, our findings may serve as the preliminary understanding of schizophrenia within the conceptual framework of cerebro-cerebellar dysconnectivity.

We found significant associations between the mean FAs in the MCP cluster and indices of executive function, verbal memory, and visuospatial organization after controlling for age, sex, and years of education. Participants with schizophrenia who had a higher mean MCP FA required more time to perform the TMT-B and produced more errors on the WCST. The mean MCP FAs were also inversely associated with poorer encoding and retrieval of verbal stimuli and visual copying. Deficits in these cognitive processes, known to be responsible for prefrontal functioning^31^, largely overlap with symptoms characterizing cerebellar cognitive-affective syndrome. Similar to motor control, the cerebellum appears to control cognitive processing by sending regulatory signals to the prefrontal cortex for its speed, consistency, capacity, and appropriateness^32^. As such, our findings suggest that aberrant WM connectivity in afferent fibers to the cerebellum from the cerebrum may adversely influence cognitive processes by interrupting adaptive abilities for organizing strategies for goal-directed achievements in patients with schizophrenia. Deficits in verbal memory may also be affected by cerebro-cerebellar dysconnectivity, as the proper use of organizational strategies is key to facilitating verbal encoding and retrieval^33^.

Our results may seem paradoxical considering that WM deficits are related to cognitive impairments in patients with schizophrenia^34,35^; however, contradictory findings have been reported in several previous studies, suggesting that higher FA does not always suggest enhanced cognitive function^15,36–38^. One possibility for our finding is that an aberrant compensatory reaction to restore WM connectivity occurred in participants with schizophrenia who demonstrated more severe WM deficits in the MCP. Since the role of the cerebellum is to produce corrective signals to minimize errors in complex sensory-motor and cognitive-affective operations, poor performance in cognitive tasks would burden the cerebellum with more conscious efforts from the cerebrum^39,40^. This overload in cognitive processing may induce an increase in myelination of the afferent WM tracts in the MCP^41^. Increased activity-dependent myelination does not necessarily appear to restore impaired cognitive function, but, rather, may be maladaptive by incurring pathological alterations in neuronal circuit dynamics^42,43^. This is partly supported by studies of traumatic brain injury showing that oligodendrocytes supporting damaged axons contribute to the formation of redundant myelin sheaths, which may lead to further damage and neuronal dysfunction^44–46^. However, future research is warranted since this study alone cannot clearly elucidate what and how biological mechanisms are involved in the observed associations between cerebro-cerebellar WM deficits and cognitive impairments in schizophrenia.

Another explanation for our findings is the disruptions to the decussation that crosses to the opposite sides of the MCP. This WM bundle contains the pontocerebellar fibers that cross the midline of the pons to enter the contralateral cerebellar hemisphere^10^. Our participants with schizophrenia showed lower FAs in the MCP than did HCs, particularly at the midline of the pons, suggesting that the decussation of the MCP is the most affected region of the cerebellar WM in schizophrenia. FA could be underestimated in WM regions rich with crossing fibers compared to regions with coherently oriented fibers^47^. Therefore, despite the overall decreased FA in participants with schizophrenia, decreased intravoxel fiber crossing caused by disrupted decussating fibers of the MCP may result in a paradoxical FA increase. This is supported by previous studies showing that FAs in crossing-fiber regions, such as the optic radiations, are inversely associated with cognitive performance, consistent with our current study^15,38,48^. Therefore, our findings are compatible with the notion of cognitive dysmetria in that impaired information transmission from the cerebral cortex to the cerebellum via the MCP may lead to deficits in executive function in patients with schizophrenia^49,50^; however, future research is warranted because the directionality of the relationship between aberrant WM connectivity of the MCP and executive function could not be determined in the current cross-sectional study.

There are several limitations that need to be considered when interpreting our findings. First, the relatively small sample sizes for each group may limit their generalization. Nonetheless, our study still provides some evidence supporting the notion that microstructural abnormalities in cerebro-cerebellar connectivity may underlie the pathogenesis of schizophrenia. Second, our cross-sectional design prevents the determination of causality of WM changes in cerebellar peduncles. Future longitudinal studies with larger samples are therefore required. Third, a portion of our participants with schizophrenia underwent DTI scan after the initiation of antipsychotic treatment. Although the mean duration of antipsychotic treatment before DTI scan was not long enough to affect WM structures (6.3 ± 6.6 days), future investigations with drug-naïve patients are required to exclude the confounding effect of antipsychotic medication. Fourth, although cognitive impairments in patients with schizophrenia is well-established^4^, we could not demonstrate how much participants with schizophrenia were impaired in cognitive function compared to HCs. Therefore, additional research with comparison data is recommended to improve our understanding of cerebro-cerebellar connectivity in relation to cognitive function in patients with schizophrenia. Finally, the issue of crossing fibers is a fundamental limitation of DTI. The future application of advanced analytic methods corrected for the crossing-fiber geometry will help address this.

In conclusion, we demonstrated disrupted WM connectivity in the MCP and its association with poorer performance in executive function in participants with schizophrenia. Our present study suggests that miscommunication between the cerebrum and cerebellum due to disrupted cerebro-cerebellar connectivity may contribute to cognitive impairments, a core characteristic of schizophrenia. Our findings may expand our understanding of the neurobiology of schizophrenia based on the cerebro-cerebellar interconnections of the brain.

## Methods

### Participants

Sixty-five participants with schizophrenia, who were antipsychotic-naïve or free of antipsychotics at least for 6 months, were recruited from the psychiatry clinic of CHA Bundang Medical Center (Seongnam, Republic of Korea), and 47 HCs were enrolled from the local community using online and print advertisements. The diagnosis of schizophrenia was made by experienced psychiatrists according to the Diagnostic and Statistical Manual for Mental Disorders, 4th edition, text revision (DSM-IV-TR) criteria using the Structured Clinical Interview for DSM-IV-TR Axis I Disorders^51^. For HCs, we included only individuals with no family history of psychiatric disorders. The exclusion criteria were as follows: (1) other psychiatric comorbidities, including mood disorders and substance-related disorders; (2) intellectual disability; (3) clinically significant or unstable medical illness; (4) neurological disorders or traumatic brain injury; and (5) any contraindications for undergoing MRI scan. We ensured that all participants were never exposed to illegal substances such as cannabis. All participants were aged 22-55 years and right-handed Koreans. Participants with schizophrenia were assessed for the severity of clinical symptoms using the Positive and Negative Syndrome Scale^52^ and Clinical Global Impression Severity of Illness^53^ at baseline.

All study procedures were reviewed and approved by the Institutional Review Board of CHA Bundang Medical Center, in accordance with the latest version of the Declaration of Helsinki and principles of Good Clinical Practice. All participants provided written informed consent following a thorough explanation of the study procedures.

### Cognitive assessments

Cognitive function was assessed using the TMT Parts A and B^54^, WCST^55^, and Rey–Kim memory test^56^ by a certified clinical psychologist in 46 participants with schizophrenia.

The purpose of the TMT is to examine the speed of processing, cognitive flexibility, cognitive set-shifting, and executive functioning^57,58^. The TMT consists of two parts (A and B), which must be performed as accurately and quickly as possible. Participants are asked to draw lines sequentially to connect 25 encircled numbers in ascending order in Part A and alternate connecting encircled numbers and letters in numerical and alphabetical order in Part B. Part A is a test of visual searching abilities and psychomotor speed, whereas Part B requires high-level cognitive skills such as cognitive flexibility concerning the additional cognitive demands of switching mental sets. The performance was measured by the total amount of time required to complete each task.

The WCST is a widely used assessment for the executive function that requires the participant to be able to abstract rules and shift cognitive strategies according to changing contingencies^59,60^. Participants are asked to match each of the response cards to one of the four stimulus cards (one red triangle, two green stars, three yellow crosses, and four blue circles) without being told a matching rule that changes after ten correct responses (i.e., color, shape, or number). Feedback on each trial was provided only to inform the participant whether their response was correct or not. The indices of performance measured were the numbers of total, perseverative, and non-perseverative errors and perseverative and conceptual level responses.

The Rey-Kim Memory Test is the Korean standardized version of the AVLT and CFT developed by Andre Rey^61–63^. The Korean version of the AVLT consists of five successive presentations of a list of 15 words followed by free recall, 20-min delayed recall, and 20-min delayed recognition trials. The number of correct words in each trial is recorded, and the scores of the five free recall trials are summed into one value. In the Korean version of the CFT, participants are presented with a complex figure and asked to copy it, followed by immediate recall and 20-min delayed recall trials. The performance was measured as the sum of scores for each of the 18 elements in the figure (maximum score = 36).

### DTI acquisition

All participants underwent MRI using a 3.0-Tesla GE Signa HDxt scanner (GE Healthcare, Milwaukee, WI, USA) comprising an eight-channel phase-array head coil at CHA Bundang Medical Center. Diffusion-weighted images were acquired using an echo planar imaging (EPI) sequence with the following parameters: repetition time = 17,000 ms; echo time = 108 ms; field of view = 240 mm; matrix = 144 × 144; slice thickness = 1.7 mm; voxel size = 1.67 × 1.67 × 1.7 mm^3^. A double-echo option was applied to minimize eddy-current-related distortions. An eight-channel coil and Array of Spatial Sensitivity Encoding Technique (GE Healthcare) with a SENSE factor of 2 were used to reduce the impact of EPI spatial distortions. Seventy axial slices parallel to the anterior commissure–posterior commissure line covering the whole brain were acquired in 51 directions with a b-value of 900 s/mm^2^. Eight baseline scans with b = 0 s/mm^2^ were also acquired. DTIs were estimated from the diffusion-weighted images using the least-squares method. The total scanning time was 17 min.

### DTI analysis

DTI data were analyzed using Functional MRI of the Brain (FMRIB) Diffusion Toolbox and Tract-Based Spatial Statistics, implemented in FMRIB Software Library (FSL version 6.0; Oxford, UK; https://fsl.fmrib.ox.ac.uk/fsl/). Preprocessing was performed according to the standard FSL protocol. We visually inspected all data for major artifacts, such as geometric distortions, signal dropouts, and insufficient image acquisition. DTIs were corrected for eddy-current- and motion-related distortions, and the b-vectors were rotated accordingly^64^. A brain mask was then created to extract brain tissue. FA images were constructed by fitting a tensor model to the corrected diffusion data and aligned in Montreal Neurologic Institute space using FMRIB’s Nonlinear Image Registration Tool. All transformed FA images were combined and applied to the original FA map, resulting in a standard-space version of the FA map. A mean FA image was created by averaging all transformed images and then skeletonized to create a mean FA skeleton representing the centers of the WM tracts (FA > 0.2). Non-FA (MD, AD, and RD) images were prepared in a similar way as provided by TBSS.

Voxel-wise comparison of FA maps between patients with schizophrenia and HCs was performed within the ROIs using permutation-based nonparametric inference within the framework of a general linear model (number of permutations = 5000). The ROI mask was created by multiplying the mean FA skeleton mask by the regional mask of the cerebellar peduncle, defined by the JHU DTI-based WM atlas^18^. Age, sex, and years of education were controlled for as covariates. The statistical significance was set at *p* < 0.05 after adjusting for multiple comparisons using the TFCE method, avoiding an arbitrary choice of the cluster-forming threshold while preserving the benefits of cluster-wise corrections.

### Statistical analysis

The demographic characteristics were compared between patients with schizophrenia and HCs using independent *t*-tests for continuous variables and chi-square tests for categorical variables. Between-group associations between cognitive performance and FAs extracted from a significant cluster were examined using partial correlations with age, sex, and years of education as covariates. The statistical significance for the partial correlations was presented uncorrected for multiple comparisons, unless otherwise indicated. All statistical procedures were carried out using the Statistical Package for the Social Sciences (version 26; IBM Corp., Armonk, NY, USA).

### Reporting summary

Further information on research design is available in the Nature Research Reporting Summary linked to this article.

## Supplementary information

Supplementary Table 1

Reporting Summary

## Data Availability

The data supporting the findings of this study are not publicly available due to ethical restrictions for protecting participants’ confidentiality and privacy but are accessible from the corresponding author on reasonable request with the approval of the Institutional Review Board of CHA Bundang Medical Center.

## Code availability

No custom code or mathematical algorithm was used in this study.
