# Supplementary material for: Impaired cerebro-cerebellar white matter connectivity and its associations with cognitive function in patients with schizophrenia
Source: NPJ Schizophr. 2021 Aug 12;7:38. doi: 10.1038/s41537-021-00169-w (PMC8360938; doi:10.1038/s41537-021-00169-w)
Supplement: Supplementary file 1 — Supplementary Table 1 [file 41537_2021_169_MOESM1_ESM.pdf]

Supplementary Table 1. Demographic and clinical characteristics of participants with schizophrenia who completed cognitive assessment and HCs

	Schizophrenia (n = 46)	HCs (n = 47)	Statistics	p value
Sex				
Male, n (%)	23 (50.0)	24 (51.1)	χ^2 = 0.04	0.835
Female, n (%)	23 (50.0)	23 (48.9)		
Age (years, mean ± SD)	34.8 ± 9.0	36.7 ± 8.6	t = -1.01	0.313
Education (years, mean ± SD)	12.9 ± 2.9	17.2 ± 2.0	t = -8.23	< 0.001
Duration of illness (months, mean ± SD)	23.6 ± 35.9			
Substance use				
Smoking, n (%)	16 (34.8)	10 (21.3)	χ^2 = 2.11	0.147
Alcohol drinking, n (%)	12 (26.1)	15 (31.9)	χ^2 = 0.38	0.536
Antipsychotic exposure				
Naïve, n (%)	41 (89.1)			
> 6 months free, n (%)	5 (10.9)			
Other psychotropic medication				
Mood stabilizer, n (%)[a]	2			
Antidepressant, n (%)[b]	2			
Duration of antipsychotics before MRI scan (days, mean ± SD)	7.2 ± 5.3			
Chlorpromazine equivalent dose of antipsychotics at MRI scan (mg/day, mean ± SD)	556.8 ± 253.1			
CGI-S (mean ± SD)	5.1 ± 1.0			
PANSS (mean ± SD)				
Positive symptom	32.1 ± 6.2			
Negative symptom	26.1 ± 9.0			
General psychopathology	64.1 ± 12.3			

HCs, healthy controls; SD, standard deviation; MRI, magnetic resonance imaging; CGI-S, Clinical Global Impression - Severity; PANSS, Positive and Negative Syndrome Scale.
[a] All participants receiving mood stabilizer were taking divalproex sodium.
[b] Antidepressants administered by participants were sertraline (n = 1) and escitalopram (n = 1).
