# Supplementary material for: Impaired cerebro-cerebellar white matter connectivity and its associations with cognitive function in patients with schizophrenia
Source: NPJ Schizophr. 2021 Aug 12;7:38. doi: 10.1038/s41537-021-00169-w (PMC8360938; doi:10.1038/s41537-021-00169-w)
Supplement: Supplementary file 2 — Reporting Summary [file 41537_2021_169_MOESM2_ESM.pdf]

nature research

Corresponding author(s): Minji Bang

Last updated by author(s): 26/04/21

Reporting Summary

Nature Research wishes to improve the reproducibility of the work that we publish. This form provides structure for consistency and transparency in reporting. For further information on Nature Research policies, see our Editorial Policies and the Editorial Policy Checklist.

Statistics

For all statistical analyses, confirm that the following items are present in the figure legend, table legend, main text, or Methods section.

n/a	Confirmed	
☐	☒	The exact sample size (n) for each experimental group/condition, given as a discrete number and unit of measurement
☐	☒	A statement on whether measurements were taken from distinct samples or whether the same sample was measured repeatedly
☐	☒	The statistical test(s) used AND whether they are one- or two-sided<br>*Only common tests should be described solely by name; describe more complex techniques in the Methods section.*
☐	☒	A description of all covariates tested
☐	☒	A description of any assumptions or corrections, such as tests of normality and adjustment for multiple comparisons
☐	☒	A full description of the statistical parameters including central tendency (e.g. means) or other basic estimates (e.g. regression coefficient) AND variation (e.g. standard deviation) or associated estimates of uncertainty (e.g. confidence intervals)
☐	☒	For null hypothesis testing, the test statistic (e.g. F, t, r) with confidence intervals, effect sizes, degrees of freedom and P value noted<br>*Give P values as exact values whenever suitable.*
☒	☐	For Bayesian analysis, information on the choice of priors and Markov chain Monte Carlo settings
☐	☒	For hierarchical and complex designs, identification of the appropriate level for tests and full reporting of outcomes
☐	☒	Estimates of effect sizes (e.g. Cohen's d, Pearson's r), indicating how they were calculated

Our web collection on statistics for biologists contains articles on many of the points above.

Software and code

Policy information about availability of computer code

Data collection	No software was used in data collection.
Data analysis	Functional MRI of the Brain (FMRIB) Diffusion Toolbox and Tract-Based Spatial Statistics (TBSS), implemented in the FMRIB Software Library (FSL version 6.0; Oxford, UK; https://fsl.fmrib.ox.ac.uk/fsl/)<br>SPSS version 26 (IBM Corporation; Armonk, NY, USA)

For manuscripts utilizing custom algorithms or software that are central to the research but not yet described in published literature, software must be made available to editors and reviewers. We strongly encourage code deposition in a community repository (e.g. GitHub). See the Nature Research guidelines for submitting code & software for further information.

Data

Policy information about availability of data

All manuscripts must include a data availability statement. This statement should provide the following information, where applicable:

- Accession codes, unique identifiers, or web links for publicly available datasets
- A list of figures that have associated raw data
- A description of any restrictions on data availability

The data supporting the findings of this study are not publicly available due to ethical restrictions for protecting participants' confidentiality and privacy but are accessible from the corresponding author on reasonable request with the approval of the Institutional Review Board of CHA Bundang Medical Center.

Field-specific reporting

Please select the one below that is the best fit for your research. If you are not sure, read the appropriate sections before making your selection.

☒ Life sciences ☐ Behavioural & social sciences ☐ Ecological, evolutionary & environmental sciences

For a reference copy of the document with all sections, see nature.com/documents/nr-reporting-summary-flat.pdf

Life sciences study design

All studies must disclose on these points even when the disclosure is negative.

Sample size	A total of 112 participants (65 patients with schizophrenia and 47 healthy controls) were included in this study. The sample size was not calculated since this study was conducted in an exploratory way.
Data exclusions	The exclusion criteria were as follows: (1) other psychiatric comorbidities, including mood disorders and substance-related disorders; (2) intellectual disability; (3) clinically significant or unstable medical illness; (4) neurological disorders or traumatic brain injury; and (5) any contraindications for undergoing magnetic resonance imaging scan.
Replication	The results have not been replicated in independent samples.
Randomization	Randomization was not relevant to this study since it was a clinical case-control design that compared patients and healthy controls.
Blinding	Blinding was not relevant to this study since it was an observational study.

Reporting for specific materials, systems and methods

We require information from authors about some types of materials, experimental systems and methods used in many studies. Here, indicate whether each material, system or method listed is relevant to your study. If you are not sure if a list item applies to your research, read the appropriate section before selecting a response.

Materials & experimental systems

n/a	Involved in the study	
☒	☐	Antibodies
☒	☐	Eukaryotic cell lines
☒	☐	Palaeontology and archaeology
☒	☐	Animals and other organisms
☐	☒	Human research participants
☒	☐	Clinical data
☒	☐	Dual use research of concern

Methods

n/a	Involved in the study	
☒	☐	ChIP-seq
☒	☐	Flow cytometry
☐	☒	MRI-based neuroimaging

Human research participants

Policy information about studies involving human research participants

Population characteristics	Sixty-five antipsychotic-naive or -free patients with schizophrenia, diagnosed based on the Diagnostic and Statistical Manual of Mental Disorders, Fourth Edition, Text Revision (DSM-IV-TR), and 47 healthy controls (HCs) participated in this study. All participants were aged between 22 and 55 years. Fifty-six (86.2%) of the 65 participants with schizophrenia were recent-onset patients who had developed psychotic symptoms within the last 5 years. There were no significant differences between participants with schizophrenia and HCs in demographic variables except for years of education.
Recruitment	Participants with schizophrenia were recruited from the psychiatry clinic of CHA Bundang Medical Center (Seongnam, Republic of Korea), and 47 healthy controls (HCs) were enrolled from the local community using online and print advertisements. For HCs, we included only individuals with no family history of psychiatric disorders. The exclusion criteria were as follows: (1) other psychiatric comorbidities, including mood disorders and substance-related disorders; (2) intellectual disability; (3) clinically significant or unstable medical illness; (4) neurological disorders or traumatic brain injury; and (5) any contraindications for undergoing magnetic resonance imaging (MRI) scan.
Ethics oversight	All study procedures were reviewed and approved by the Institutional Review Board of CHA Bundang Medical Center, in accordance with the latest version of the Declaration of Helsinki and principles of Good Clinical Practice.

Note that full information on the approval of the study protocol must also be provided in the manuscript.

Magnetic resonance imaging

Experimental design

Design type	N.A.
Design specifications	N.A.
Behavioral performance measures	N.A.

Acquisition

Imaging type(s)	Diffusion tensor imaging (DTI)
Field strength	3 Tesla
Sequence & imaging parameters	Diffusion-weighted images (DWI) were acquired using an echo planar imaging (EPI) sequence with the following parameters: repetition time, 17,000 ms; echo time, 108 ms; field of view, 240 mm; matrix, 144 × 144; slice thickness, 1.7 mm; and voxel size, 1.67 × 1.67 × 1.7 mm3. A double-echo option was applied to minimize eddy-current-related distortions. An eight-channel coil and Array of Spatial Sensitivity Encoding Techniques (GE Healthcare) with a SENSE factor of 2 were used to reduce the impact of EPI spatial distortions. Seventy axial slices parallel to the anterior commissure–posterior commissure line covering the whole brain were acquired in 51 directions with a b-value of 900 s/mm2. Eight baseline scans with b = 0 s/mm2 were also acquired. The total scanning time was 17 min.
Area of acquisition	Whole-brain images were acquired.

Diffusion MRI ☒ Used ☐ Not used

Parameters: See above.

Preprocessing

Preprocessing software	Functional MRI of the Brain (FMRIB) Diffusion Toolbox, implemented in the FMRIB Software Library (FSL version 6.0; Oxford, UK; https://fsl.fmrib.ox.ac.uk/fsl/)
Normalization	Diffusion tensor images underwent FSL's standard preprocessing steps including skull stripping and eddy-current correction. Fractional anisotropy (FA) images were created by fitting a tensor model to the corrected diffusion data and then aligned in the Montreal Neurologic Institute standard space.
Normalization template	Montreal Neurologic Institute standard space
Noise and artifact removal	Skull tripping and eddy-current correction were preformed.
Volume censoring	N.A.

Statistical modeling & inference

Model type and settings	Statistical analysis of DTI data was performed using permutation-based non-parametric inference within the framework of a general linear model.
Effect(s) tested	Comparison for FAs between patients with schizophrenia and healthy controls (t-test)

Specify type of analysis: ☐ Whole brain ☒ ROI-based ☐ Both

Anatomical location(s): Cerebellum WM bundles (superior, middle, and inferior cerebellar peduncles and pontine crossing fibers) were defined as regions of interest based on the Johns Hopkins University DTI-based white matter atlas.

Statistic type for inference (See Eklund et al. 2016)	Permutation-based non-parametric inference (n = 5,000) with the threshold-free cluster enhancement (TFCE) method.
Correction	TFCE method

Models & analysis

n/a	Involved in the study
☒	☐ Functional and/or effective connectivity
☒	☐ Graph analysis
☐	☒ Multivariate modeling or predictive analysis

Multivariate modeling and predictive analysis	Comparison for FAs between patients with schizophrenia and healthy controls (t-test)
